# The molecular regulation of axillary bud fate determination and outgrowth into branch crown in strawberry involves *BRC1*

**DOI:** 10.1111/nph.71375

**Published:** 2026-06-26

**Authors:** Marie Alonso, Pierre Prévost, Aline Potier, Pascal GP Martin, Yves Caraglio, Michael Nicolas, Michel Hernould, Christophe Rothan, Béatrice Denoyes, Amèlia Gaston

**Affiliations:** ^1^ Univ. Bordeaux, INRAE, Biologie du Fruit et Pathologie, UMR 1332 F‐33140 Villenave d'Ornon France; ^2^ CIRAD, UMR AMAP, and Université de Montpellier F‐34398 Montpellier France; ^3^ Plant Molecular Genetics Department Centro Nacional de Biotecnología‐CSIC, Campus Universidad Autónoma de Madrid 28049 Madrid Spain

**Keywords:** axillary bud, *Branched 1*, fate, *Fragaria vesca*, outgrowth, sexual and asexual reproduction, transcriptome

## Abstract

- In strawberry, the axillary bud (AXB) can produce either an elongated stem called stolon giving a daughter‐plant (asexual reproduction) or an inflorescence‐bearing branch crown (BC; sexual reproduction). The AXB fate depends on node position on the axis and on genetic and environmental factors. Here, in *Fragaria vesca*, we addressed the largely unanswered question of how molecular factors determine AXB fate.
- To get insights into the mechanisms already at play in a morphologically indistinguishable (undifferentiated) AXB, depending on its fate, we combined the phenotypic characterization of AXB development throughout plant growth with the RNA‐seq analysis of undifferentiated AXBs, using three different genotypes producing either BCs or stolons.
- Results allowed the identification of genes regulating AXB fate and outgrowth, among which was *FveBRC1*. Exogenous applied gibberellin failed to restore stolon formation in CRISPR/Cas9 *brc1* mutants, which produce BCs instead of stolons, thus demonstrating that *FveBRC1* is necessary for AXB determination into stolon.
- These original results provide new insights into the molecular regulation of AXB fate in strawberry and highlight the major role of *FveBRC1*, not only in repressing BC outgrowth but also in determining AXB fate.

## Graphical abstract

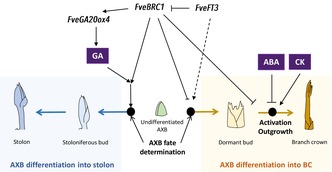

## Introduction

Plant architecture, defined as the spatial organization of the plant, has been a major domestication trait and a key target for crop improvement due to its significant impact on yield (Eshed & Lippman, 2019). It depends on the ontogenetic dynamics of both shoot apical meristem (SAM) and axillary meristems (AXM; Barthélémy & Caraglio, 2007; Labadie *et al*., 2023b). During plant growth, the SAM produces stem and initiates leaves with, in their axils, the axillary bud (AXB) containing AXM with leaf primordia. AXB can remain dormant or develop into a lateral branch along the primary shoot. In addition to lateral branches, AXB can also produce floral structures and specialized vegetative organs, including those involved in asexual reproduction, such as stolons (Wang *et al*., 2018; Guo *et al*., 2021). Stolons are diageotropic elongated stems, underground in potato where they produce tubers, aerial in strawberry where they produce daughter plants (Zierer *et al*., 2021). In cultivated strawberry (*Fragaria* × *ananassa*), a major horticultural species, the balance between stolon and branch formation is of major importance as it determines fruit and daughter‐plant yields (Tenreira *et al*., 2017). Strawberry exhibits a sympodial branching: after floral initiation under short‐day conditions the previous year, the SAM transitions to a floral meristem in spring, and the uppermost AXB of the stem (primary crown) develops into an inflorescence‐bearing branch (extension crown; Labadie *et al*., 2023b). AXBs can follow two possible fates: they can form either stolons or lateral branches (branch crowns, BC; Savini *et al*., 2008; Tenreira *et al*., 2017; Labadie *et al*., 2023a,b). Once the AXB fate is determined, the stolon develops rapidly, whereas BC development begins as a dormant bud (nonextended BC) with a dormancy period of variable length before outgrowth (Labadie *et al*., 2023a,b; Han *et al*., 2024). Unraveling the regulatory network governing AXB fate determination and outgrowth is therefore crucial for modulating the balance between sexual and asexual reproduction, ultimately impacting both fruit and plant yields (Gaston *et al*., 2021; Andrés & Koskela, 2022).

Despite its importance, how the AXB fate is determined, before subsequent differentiation into a stolon, or dormant bud and BC, has been little studied to date. In strawberry, AXB fate is governed by genetic factors and environmental conditions (Andrés *et al*., 2021; Andrés & Koskela, 2022), node position on the axis (Labadie *et al*., 2023a,b) and hormones, including gibberellins (GAs; Hytönen *et al*., 2009; Tenreira *et al*., 2017). In addition to these factors, several genetic alterations in *Fragaria vesca* can be harnessed to better understand the determinism of AXB fate in strawberry. We showed recently that the *FLOWERING LOCUS T3* (*FveFT3*) gene promotes branching when overexpressed in *F. vesca*, likely by altering AXB fate: instead of producing a stolon, the AXB develops directly into a BC, bypassing the dormancy phase before outgrowth (Gaston *et al*., 2021). *FveFT3* is a member of the CENTRORADIALIS/TERMINAL FLOWER 1/SELF‐PRUNING (CETS) family (FT/TFL1) primarily known for its crucial role in flowering, with FT as a florigen and TFL1 as a floral repressor (Wickland & Hanzawa, 2015; Gaston *et al*., 2021). The interplay between FT and TFL1 is essential in shaping architecture (Moraes *et al*., 2019; Colleoni *et al*., 2024), including in strawberry (Gaston *et al*., 2021; Andrés & Koskela, 2022). In *F. vesca*, a natural mutation in *FveTFL1* leads to perpetual flowering (PF), which is the ability to flower continuously along vegetative development through the sustained production of new BCs (Iwata *et al*., 2012; Koskela *et al*., 2012). Two other mutations influence AXB fate, a mutation in the *FveGA20ox4* GA biosynthesis gene responsible for the natural runnerless (no stolon) phenotype (Tenreira *et al*., 2017; Andrés *et al*., 2021), whereas a mutation in the GA signaling gene, *FveRGA1* (*REPRESSOR OF GIBBERELLIC ACID1*) of the *DELLA* family, reversing the effect of the *fvega20ox4* mutation (Caruana *et al*., 2018; Li *et al*., 2018). Recently, the zinc‐finger protein, *FveZFP6*, has been identified as an upstream regulator of stolon fate determination by activating GA biosynthesis (Guo *et al*., 2026). Despite these findings, the regulatory network and molecular mechanisms controlling AXB fate determination in strawberry remains largely unexplored.

The regulation of AXB outgrowth into lateral branches in plants is comparatively better documented. AXB outgrowth has been well studied in species producing only lateral branches, where AXB activation from dormancy to lateral branch outgrowth is mainly controlled by the apical dominance exerted by SAM (Cline, 1997; Barbier *et al*., 2019) and crosstalk between the plant hormones auxin, cytokinin, strigolactone and abscisic acid (Ferguson & Beveridge, 2009; Wang *et al*., 2018; Barbier *et al*., 2019). Stolon‐bearing species are more complex to study as AXB outgrowth results in either stolon or BC. In strawberry, the GATA transcription factor *HANABA TARANU* (*FaHAN*) and the GRAS transcription factor LOSS AXILLARY MERISTEMS (*FveLAM*) have been identified as key regulators of AXM initiation and AXB outgrowth into stolon (Feng *et al*., 2021; Liang *et al*., 2022). Conversely, *FveMYB117a and FveDAD2* have been shown to inhibit AXB outgrowth into BC by repressing the cytokinin pathway or enhancing the abscisic acid (ABA) pathway via strigolactone signaling (Han *et al*., 2024; Sun *et al*., 2025). The regulatory network controlling AXB outgrowth into a lateral branch converges on a major repressor which interacts with FT/TFL1, the *TEOSINTHE BRANCHED 1/CYCLOIDEA/PROLIFERATING CELL FACTOR* (*TCP*() transcription factor, *BRANCHED1* (*BRC1*); Aguilar‐Martínez *et al*., 2007; Wang *et al*., 2019; Colleoni *et al*., 2024). Based on the known role of *BRC1* in AXB dormancy and outgrowth into lateral branches, and its expression pattern in strawberry, the closest *F. vesca* homolog of *AtBRC1*, *FveTCP9* (hereafter referred to as *FveBRC1*), was hypothesized to play a role in AXB outgrowth into BC (Wei *et al*., 2016; Qiu *et al*., 2019; Han *et al*., 2024).

Here, we reasoned that analyzing undifferentiated AXBs while considering their subsequent fate would provide a comprehensive understanding of the molecular mechanisms of AXB fate determination. To this end, we first characterized early AXB development in *F. vesca* to establish a developmental scale describing the stages of AXB outgrowth. We then used this scale to examine the spatio‐temporal growth of AXBs along the primary crown according to their subsequent fates, stolon or BC. Next, we performed a transcriptome analysis of morphologically undifferentiated AXBs with distinct predicted fates to uncover early molecular signatures underlying AXB determination. Finally, we investigated *FveBRC1*, highlighted through transcriptome analysis, revealing its pivotal and original role in AXB fate determination, in addition to its already known role in AXB activation and BC outgrowth. These findings make *FveBRC1* a promising target for improving cultivated strawberries.

## Materials and Methods

### Plant material and growth conditions

Diploid strawberry (*Fragaria vesca* L.) genotypes were used in this study. Seeds were sown in a mixture of two‐thirds loam and one‐third grit and grown in long‐day conditions (16 h : 8 h, 22°C : 18°C, day : night, at; 250 μmol m^−2^ s^−1^). Plants were transplanted into 1‐liter pots containing the same mixture and were maintained in a glasshouse under natural conditions. Three genotypes carrying the *tfl1* mutation that produces the PF phenotype (Iwata *et al*., 2012) were used for studying AXB development and plant architecture as well as for transcriptome analysis. These genotypes included: ‘Hawaii‐4’ (H4); the *35S::FveFT3FveOE* (*FT3* ^*OE*^) transgenic line, which overexpresses *FveFT3* in the H4 genetic background (Gaston *et al*., 2021); and the runnerless [r]/[PF] ‘Reine des Vallées’ (RdV) variety, which is a *FveGA20ox4/FveTFL1* mutant (Tenreira *et al*., 2017). H4 was used for gene editing of the *FveBRC1* gene with the CRISPR/Cas9 system (CR‐*fvebrc1* mutants).

### Plant dissection for the determination of AXB development and plant architecture

Strawberry AXBs were observed by dissecting 2 to 4‐month‐old seedlings using a stereomicroscope (SZX16; Olympus, Tokyo, Japan). The AXB developmental scale was established by analyzing the spatio‐temporal development of AXBs using photographs obtained with the EPview software driving an Olympus EP50 camera mounted on the stereomicroscope. Each description of an AXB included its developmental stage and node position (Labadie *et al*., 2023b). The plant developmental stage was determined according to the number of trifoliate leaves developed plus the new, nonexpanded leaf (Supporting Information Fig. S1). Patterns of AXB development were determined on 10 plants per genotype, starting from the three‐leaf stage up to flowering. For CR‐*fvebrc1* mutants, the number of trifoliate leaves, crowns, stolons and inflorescences was counted on 2‐ to 18‐month‐old primary transformants. Architecture of all genotypes was described after dissecting plants as described in Gaston *et al*. (2021).

### Histological studies and *in situ* hybridization

For histological studies and *in situ* hybridization, longitudinal sections of the primary crown were prepared from three to five trifoliate leaf seedlings after leaf removal. Dissected samples were prepared as described in Tenreira *et al*. (2017). For *in situ* hybridization of *FveBRC1*, undifferentiated AXBs were collected from 3‐month‐old plants of the H4, *FT3* ^*OE*^ and RdV genotypes. ISH‐*FveBRC1* primers used for synthesizing digoxygenin‐UTP‐labelled sense and antisense RNA probes are provided in Table S1.

### RNA‐seq and qPCR analyses

For RNA‐seq and qPCR analysis, undifferentiated AXBs were collected at the fourth node of four‐leaf stage plants (which represents three trifoliate leaves developed plus a new, nonexpanded leaf) on the newly nonexpanded leaf on studied genotypes, immediately frozen in liquid nitrogen and stored at −80°C until analysis. Each genotype was represented by three biological replicates. Each sample consisted of 30 undifferentiated AXB comprising one AXM, leaf primordium, surrounded by two prophylls collected on 30 plants. Total RNA was extracted as described in Gaston *et al*. (2021). For RNA‐seq analysis on H4, *FT3* ^*OE*^ and RdV genotypes, stranded mRNA‐seq libraries were prepared and sequenced (150‐bp paired‐end reads) by GenoToul (Toulouse, France). The *F. vesca* v4.0.a1 genome (Edger *et al*., 2017) and its v4.a2 annotation (Li *et al*., 2019b) were used as reference. Reads were mapped using Star v.2.7.9a (Dobin *et al*., 2013) with default parameters. Gene level draw counts and Transcripts per Million (TPM) values were obtained with Rsem (v.1.3.3; Li & Dewey, 2011). Differential expression was analyzed using the ‘exactTest()’ function of the R/Bioconductor edgeR package (v.3.32.1; R Core Team, 2025; Robinson *et al*., 2010) following Relative Log Expression (RLE) normalization and multiple testing correction with the Benjamini–Hochberg procedure. Gene expression changes were considered significant using a cutoff of false discovery rate (FDR) 0.05 and an absolute log_2_ (fold change) greater than 1. Exploratory analysis was conducted using the R software (v.3.6.1). Principal component analysis (PCA) was performed on TPM values using the 500 most variable genes with the ‘factominer (v.2.4)’ (Lê *et al*., 2008) and factoextra (v.1.0.7) (Kassambara & Mundt, 2020) packages. Venn diagrams were created using the Venny v.2.1.0 platform (BioinfoGP, CNB‐CSIC). Hierarchical clustering of genes differentially expressed in at least one comparison and heatmaps were obtained from *z*‐scores calculated from TPM values using enrichedheatmap (v.1.16.0) package (Gu *et al*., 2018). Fisher's exact tests (R package topgo v2.38.1, Alexa & Rahnenfuhrer, 2025) were used to identify the Gene Ontology (GO) Biological Processes enriched in each cluster. Gene Set Enrichment Analysis (GSEA) was carried out with the clusterprofiler package (v4.12.6; Yu *et al*., 2012). The ‘Dormancy’, ‘Bud activation’, ‘BRC1up’ and ‘BRC1down’ genes sets were previously described (Tarancón *et al*., 2017; Nicolas *et al*., 2022). Parameters for studying the overrepresentation of the different gene sets were FDR for method and 0.5 for *P*valuecutoff. Quantitative reverse transcription polymerase chain reaction was performed following the protocol described in Gaston *et al*. (2021). Primers are provided in (Table S1).

### Plasmid constructs and plant transformation

CRISPR/Cas9 (Clustured Regurlarly Interspaced Short Palindromic Repeats/CRISP‐R‐associated protein 9) mutagenesis of *FveBRC1* using the H4 genotype was carried out with two sgRNAs (Table S1) as described in Gaston *et al*. (2021). Resulting mutations were detected by sequencing T1 lines. Plants carrying four independent homozygous mutations were selected for further analysis.

### GA3 treatment

For GA3 treatments, 5‐month‐old H4, RdV, *FT3* ^*OE*^ and T2 homozygous CR‐*fvebrc1* plants were sprayed twice a week for two consecutive weeks with either GA_3_ (50 mg l^−1^) or mock solution following the protocol described in Tenreira *et al*. (2017). Five plants per genotype and per treatment condition were analyzed. Plants were phenotyped 1 month after the onset of the treatment.

### Statistical analyses

Kruskal–Wallis test (ANOVA) followed by a Tukey *post hoc* test was carried out on *CR‐fvebrc1* gene expression results. Wilcoxon‐Mann–Whitney test was used for phenotypic analysis to compare the number of leaves, stolons, BCs and inflorescences in WT and *CR‐fvebrc1* mutants.

## Results

### Establishment of an AXB developmental scale

**Fig. 1:**
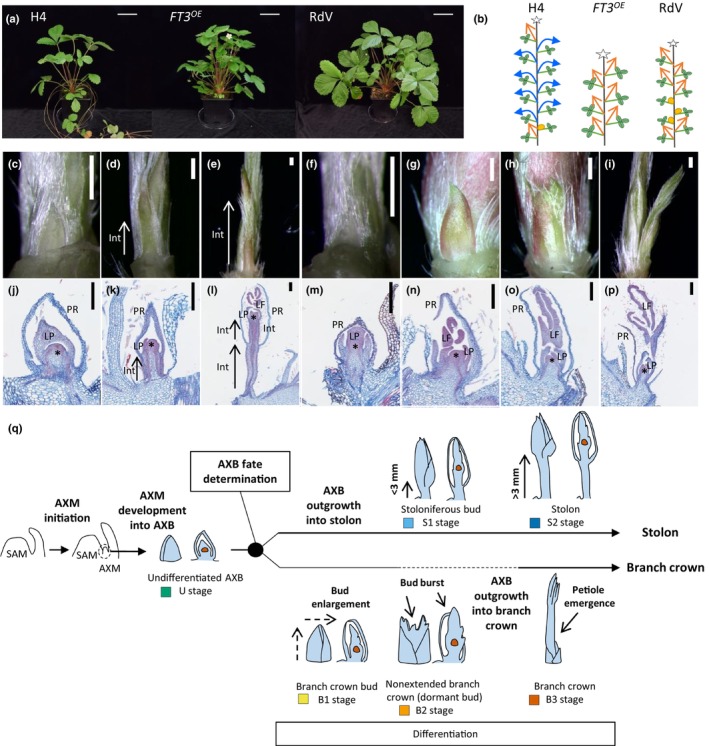
Developmental stages of the axillary bud (AXB) fate determination and differentiation into stolon or branch crown (BC) in strawberry. (a) ‘Hawaii‐4’ (H4), *35S::FveFT3* ^*FveOE*^ (*FT3* ^*OE*^) and ‘Reine des Vallées’ (RdV) 4‐month‐old plants. Bars, 5 cm. (b) Schematic plant architecture of the primary crown of each genotype. Each primary crown (green vertical line) is terminated by an inflorescence (white star). The AXB in the axil of each leaf can differentiate into a stolon (blue arrow), a branch crown (BC; orange arrow) or remain dormant (orange circle). (c–i) Morphological characterization of AXB differentiation into a stolon or a BC. Bars, 500 μm. (c–e) Differentiation of AXB into a stolon in H4. The white arrow indicates the first internode (Int), which elongates in (d) stoloniferous bud and (e) stolon. (f–i) Differentiation of AXB into a BC in *FT3* ^*OE*^. (j–p) Histological characterization of AXB differentiation into stolon or BC. AXB sections were stained with astra bleu and safranin for differentiated and dividing cells, respectively. The AXB, surrounded by prophylls (PR), shows a meristematic dome (black asterisk) at its center and developing leaf primordia (LP). LP develop into leaves (LF, leaf). In AXB differentiating into stolon (j–l), the black arrow in (k) indicates the first internode (Int), the elongation of the stoloniferous bud and (l) the elongation of the two first internodes of the stolon. (m–p) AXB differentiation into a BC. Bars, 200 μm. (q) AXB development scale starting with axillary meristem (AXM) initiation, followed by AXM development into undifferentiated AXB (U stage), and AXB decision to differentiate into either a stolon or a BC (AXB fate determination). The AXB differentiation into stolon is characterized by the elongation of the first internode, defined as the stoloniferous bud stage (S1, elongation < 3 mm), which is followed by the stolon stage (S2, elongation > 3 mm). The differentiation of AXB into BC is characterized by the enlargement of the AXB (B1 stage), followed by a variable period of dormancy (dormant bud or nonextended BC, B2 stage), and by AXB outgrowth into BC that is characterized by petiole emergence (B3 stage). Each stage of AXB development is characterized by a different color: green, undifferentiated AXB (U); pale blue, stoloniferous bud (S1); blue, stolon (S2); yellow, BC bud (B1); orange, dormant bud or nonextended BC (B2); dark orange, branch crown BC (B3).

To provide a detailed description of the early stages of AXB development, three genotypes producing either stolons (‘Hawaii‐4’ (H4)) or BCs (*FT3* ^*OE*^ and ‘Reine des Vallées’ (RdV); Fig. 1a,b) were characterized at morphological (Fig. 1c–i) and histological (Fig. 1j–p) levels. In all three genotypes, the AXB in the axil of the newly nonexpanded leaf (comprising AXM with one or two leaf primordia protected by two prophylls) was triangular in shape (H4 and *FT3* ^*OE*^; Fig. 1c,j,f,m, and RdV; Fig. S2). Elongation of the first internode occurred first at the stoloniferous bud apex (Fig. 1k). Later in stolon development, we observed the elongation of the second internode and the development of leaves in the daughter plant (Fig. 1l). In *FT3* ^*OE*^ and RdV, the differentiation into BC was associated with the thickening of the AXB (Figs 1g–n, S2). Then, the first leaf grew beyond the top of the prophylls (Fig. 1h–o) until the leaf was fully developed (Fig. 1i,p).

These observations were used to establish a scale for AXB development (Fig. 1q). The first stage was observed in the axil of the newly nonexpanded leaf. At this stage, AXBs were morphologically indistinguishable regardless of their final fate, stolon or BC. We have therefore called this stage the undifferentiated stage (U stage), during which the two different developmental fates (stolon or BC) are likely already determined. For the AXB differentiating into stolon, we set a threshold for first internode elongation at 3 mm, below which the AXB was considered as a stoloniferous bud (S1 stage) and above which it was considered as a stolon (S2 stage). When the AXB produced a BC, the thickened bud was named BC bud (B1 stage). When the bud burst and the leaf began to be visible, we considered AXB to be a nonextended BC (dormant bud, stage B2); the emergence of a leaf petiole from the BC bud indicated the BC development (B3 stage).

### AXB fate depends on its node position on the primary crown and the genotype

**Fig. 2:**
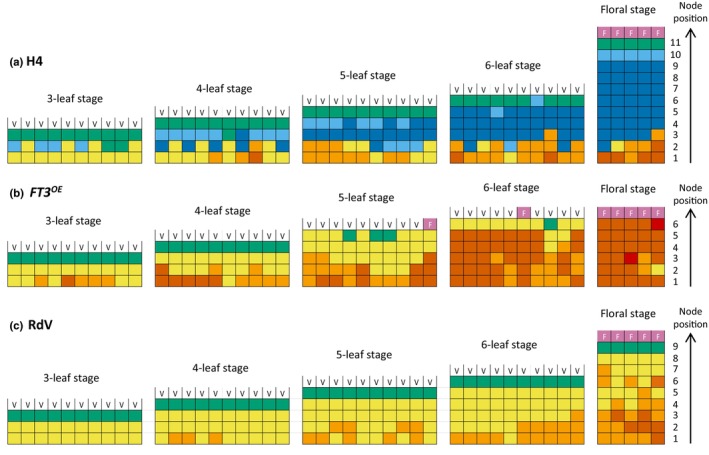
Patterns of axillary bud (AXB) development from the vegetative stage to flowering in (a) ‘Hawaii‐4’ (H4), (b) *35S::FveFT3* ^*FveOE*^ (*FT3* ^*OE*^) and (c) ‘Reine des Vallées’ (RdV) strawberry plants. The architecture of ‘Hawaii‐4’ (H4), *35S::FveFT3* ^*FveOE*^ (*FT3* ^*OE*^) and ‘Reine des Vallées’ (RdV) plants was determined by dissection of plants at the three‐ to six‐leaf stages (*n* = 10) to the flowering stage (*n* = 5). At a given stage (X), there are X‐1 developed leaves and one emerging leaf (nonexpanded leaf). Each column represents a single plant. Each square box represents a node, including the AXB. Box colors correspond to the stages of AXB development: green, undifferentiated AXB (U); pale blue, stoloniferous bud (S1); blue, stolon (S2); yellow, BC bud (B1); orange, dormant bud or nonextended branch crown (B2); dark orange, branch crown BC (B3). Purple box, floral BC. V, vegetative apical meristem; F, floral apical meristem.

To characterize the spatio‐temporal dynamics of AXB outgrowth into a stolon or BC, we determined the plant architecture of the three genotypes, H4, *FT3* ^*OE*^ and RdV, starting from the three‐leaf stage (i.e. two developed leaves plus a new, nonexpanded leaf) to the six‐leaf and floral stages (Fig. 2). We then scored AXB stages at each node on the primary crown, according to the established scale (Fig. 1q). For the three genotypes, undifferentiated AXBs were observed only in the axil of nonexpanded leaves, which corresponded to the last‐emerged node. Below this point, AXBs were already differentiated, displaying a gradient of developmental stages, with those closer to the SAM exhibiting a less advanced stage.

The AXB fate was dependent on the genotype. In the H4 genotype, AXBs at Nodes 3 to 9 developed into stolons (S2 stage; Figs 1b, 2a) while AXBs of the first two nodes could eventually produce BCs. In the *FT3* ^*OE*^ and RdV genotypes, all AXBs differentiated into BC buds (B1 stage; Figs 1b, 2b,c). AXB development into BCs was rapid in *FT3* ^*OE*^, with the production of vegetative branches (B3) starting at the three‐leaf stage. In RdV, AXB outgrowth progressed more slowly, reaching the B2 stage; some B2 AXBs, particularly those located at Nodes 1 to 3, could develop into BCs (B3 stage) after a variable period of dormancy (Fig. 2b,c).

Flowering time was also variable between the genotypes. In RdV and H4, flowering took place at the 9‐ and 11‐leaf stages, respectively, while *FT3* ^*OE*^ flowered earlier, at the six‐leaf stage (Fig. 2). In RdV and H4, the uppermost AXB of the primary crown produced an inflorescence‐bearing branch (extension crown) after flowering (Fig. 2a,c), thus enabling sympodial branching (Fig. 1b). Remarkably, thanks to the detailed characterization of the temporality of plant development for the three genotypes (Fig. 2), we were able to predict with great confidence the AXB fate while they were still undifferentiated. From the fourth to the penultimate AXB, we could determine that the final AXB fate was the production of a stolon in H4 and BC in RdV and *FT3* ^*OE*^, with or without an intermediate step of dormancy, respectively.

### Transcriptome analysis of undifferentiated AXBs provides comprehensive pictures of the determination of AXB fate and regulation of branch crown formation by *FveFT3*

**Fig. 3:**
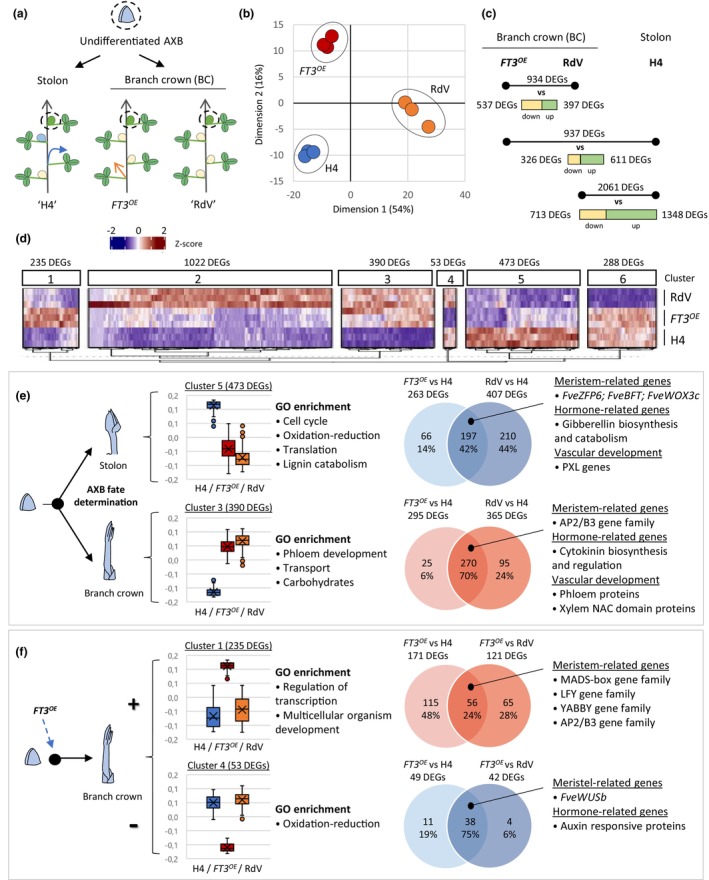
Differential gene expression analysis between undifferentiated axillary buds (AXBs) of ‘Hawaii‐4’ (H4), *35S::FveFT3* ^*FveOE*^ (*FT3* ^*OE*^) and ‘Reine des Vallées’ (RdV) strawberry genotypes. (a) RNA‐seq design. Dotted circles represent the undifferentiated AXBs collected from Node 4 of plants at the four‐leaf stage of the three genotypes H4, *FT3* ^*OE*^ and RdV. AXBs of H4 differentiate into a stolon while AXBs of *FT3* ^*OE*^ and RdV differentiate into a branch crown (BC). (b) Principal component analysis (PCA) plot of the 500 genes with the most variable expression. (c) Total number of significant differentially expressed genes (DEGs) in each pairwise comparison (*FT3* ^*OE*^ vs RdV, *FT3* ^*OE*^ vs H4 and RdV vs H4). Green and yellow bars indicate the number of upregulated and downregulated genes, respectively, for each pairwise comparison. (d) Hierarchical clustering analysis (HCA) of the 2461 DEGs identified in at least one pairwise comparison between the three genotypes. Each column represents the normalized expression values (*z*‐score) of a transcript for each sample in a row (including triplicates). Red indicates a high transcript abundance, blue low. Six clusters were identified according to the degree of similarity of the expression profile of each gene for the three genotypes. (e) DEGs from Cluster 3 and 5 involved in the determination of AXB fate and (f) DEGs from Cluster 1 and 4 involved in the regulation by *FveFT3* of AXB differentiation into BC and BC outgrowth. Boxplots show gene expression patterns (triplicates average values of *z*‐scores) for the three genotypes. Selected representative Gene Ontology (GO) terms enriched in each cluster are listed. Venn diagrams represent the number of DEGs and common DEGs in the pairwise comparisons indicated. Selected categories of genes enriched in common DEGs are listed.

To provide evidence for the early molecular programs implemented in the undifferentiated AXB, we collected undifferentiated AXBs with known fates (stolon or BC, with or without AXB dormancy) at Node 4 on four‐leaf stage plants of the three genotypes studied (Fig. 3a) and subjected them to transcriptome analyses. PCA enabled us to clearly discriminate between the three genotypes (Fig. 3b). The first two dimensions (PC1 and PC2) explained 54% and 16% of the structural variance, respectively. PC1 clearly separated RdV from H4 and *FT3* ^*OE*^, thus highlighting the strong effect of the genetic background. PC2 allowed the distinction of all three genotypes with a clear separation between H4 and *FT3* ^*OE*^. Both share the same genetic background, indicating the strong effect of *FveFT3* overexpression on gene expression as early as the undifferentiated AXB stage.

To investigate at the molecular level, the determination of AXB fate, regulation of BC differentiation and development by *FveFT3* and induction of dormancy, we next performed pairwise comparisons of differentially expressed genes (DEGs) between AXBs of the three genotypes. Comparison of AXB transcriptomes revealed DEGs between genotypes with (i) a different AXB fate and the same genetic background (*FT3* ^*OE*^ vs H4; 937 DEGs), (ii) the same AXB fate but a different genetic background (*FT3* ^*OE*^ vs RdV; 934 DEGs) and (iii) a different AXB fate and genetic background (RdV vs H4; 2061 DEGs; Table S2; Figs 1b, 3c). The highest change in the total number of DEGs was observed between H4 and RdV, which is consistent with their different genetic background and AXB fate (Fig. 3c).

To identify groups of genes sharing similar expression patterns, we performed hierarchical clustering of the 2461 DEGs identified in at least one pairwise comparison and identified six clusters (C1 to C6; Fig. 3d; Table S2). In Clusters 3 and 5 (390 and 473 DEGs, respectively), the transcript profile of H4 (stolon) is opposite to those of *FT3* ^*OE*^ and RdV (BC), suggesting clusters involved in AXB fate determination. In Clusters 1 and 4 (235 and 53 DEGs, respectively), the transcript profile of *FT3* ^*OE*^ is opposite to those of RdV and H4, suggesting a specific regulation of AXB differentiation by *FveFT3*. In Clusters 2 and 6 (1023 and 288 DEGs, respectively), the transcript profile of RdV (dormant AXBs) is opposite to those of H4 and *FT3* ^*OE*^ (nondormant AXBs), suggesting gene expression patterns associated with AXB dormancy; although, an effect of the genetic background (RdV vs H4/*FT3* ^*OE*^) cannot be ruled out.

### Identification of key genes involved in the AXB fate determination

**Fig. 4:**
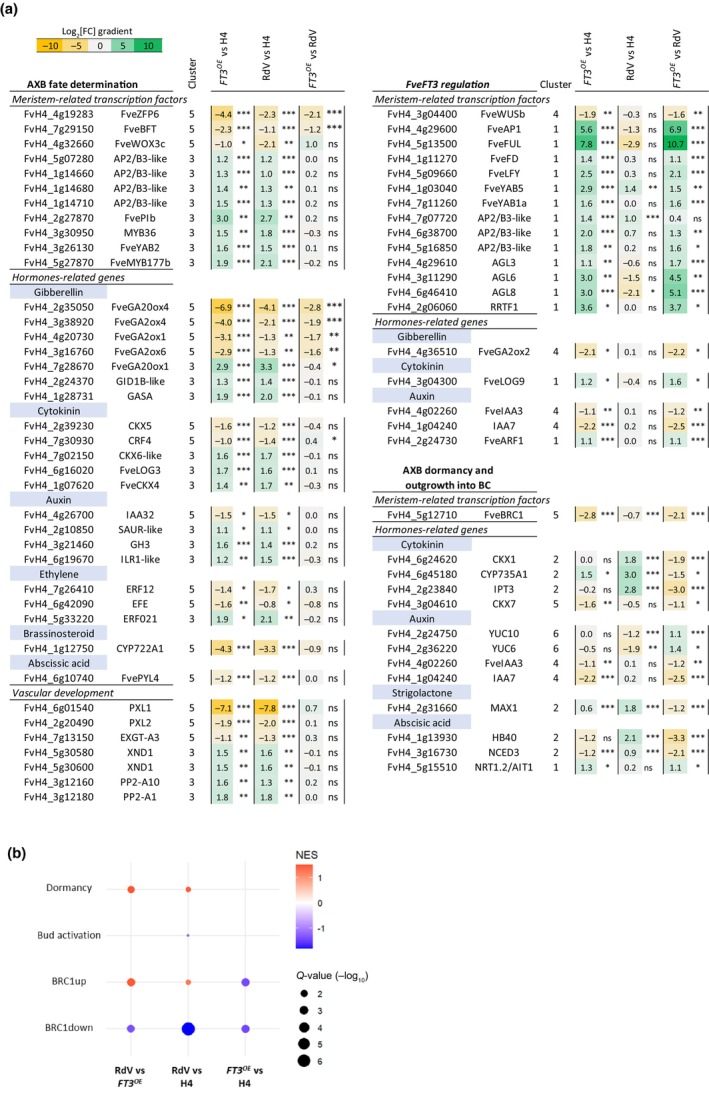
Axillary bud (AXB) fate, dormancy and outgrowth into branch crown (BC) in strawberry. (a) Selected differentially expressed genes (DEGs) associated with AXB fate, regulation by *FveFT3* and AXB dormancy and outgrowth into BC as determined by RNA‐seq analysis of undifferentiated AXBs of ‘Hawaii‐4’ (H4), *35S::FveFT3* ^*FveOE*^ (*FT3* ^*OE*^) and ‘Reine des Vallées’ (RdV) genotypes. The three different analyses (‘AXB fate’, ‘*FveFT3* regulation’ and ‘AXB dormancy and outgrowth into BC’) are shown in bold. For example, ‘*FT3* ^*OE*^ vs H4’ indicates genes that are downregulated (yellow) or upregulated (green) in *FT3* ^*OE*^ relative to H4. Each analysis is subdivided into categories (in italics). The ‘hormone‐related genes’ category is further divided into sections corresponding to different hormones (highlighted in blue). The assignment of each gene to a cluster is indicated. The heat map shows log2[FC] values for each comparison. Statistical significance levels are indicated as follows: ns, not significant; *, *P* < 0.05; **, *P* < 0.01; ***, *P* < 0.001. (b) Gene Set Enrichment Analysis (GSEA) was performed for three pairwise comparisons: RdV vs *FT3* ^*OE*^, RdV vs H4, *FT3* ^*OE*^ vs H4 using four gene sets: dormancy, bud activation, BRC1 dependent upregulated genes (BRC1up) and BRC1‐dependent downregulated genes (BRC1down). Only gene sets showing statistically significant enrichment with a *P*‐value below the cutoff (0.5 for *P*valuecutoff) are displayed, with dot size proportional to the q‐value and color representing the normalized enrichment score (NES).

To identify genes positively associated with AXB determination into stolon, we examined the 473 DEGs in Cluster 5, which exhibited higher transcript levels in H4 (stolon) than in *FT3* ^*OE*^ and RdV (BC). GO enrichment analysis indicated that it was enriched in morphogenesis‐related terms likely reflecting early cell growth processes such as ‘protein translation’ and ‘cell cycle’ as well as later specialization, such as ‘photosynthesis’, ‘anatomical structure development’ and ‘lignin catabolism’ (Fig. 3e; Table S3). Interestingly, we found three *TCP* genes: *FveBRC1*, the closest homolog to *AtBRC1*, a major repressor of shoot branching by promoting AXB dormancy (Aguilar‐Martínez *et al*., 2007; Wang *et al*., 2019), and *FveTCP18* and *FveTCP11*, belonging to the PCF clade (Fig. S3). Among the 473 Cluster 5 DEGs, we then focused on the 197 DEGs common to the *FT3* ^*OE*^ vs H4 and RdV vs H4 comparisons (Fig. 3e). These 197 DEGs strictly associated with AXB determination into stolon were involved in ‘transcriptional regulation’, ‘hormone biosynthesis and signaling’, and ‘vascular development’ (Table S3). These 197 DEGs included the meristem‐related genes ZINC‐FINGER PROTEIN 6 (*FveZFP6*), *BROTHER OF FT AND TFL1* (*FveBFT*) and *WUSCHEL‐related HOMEOBOX3c* (*FveWOX3c*); a set of genes involved in hormone biosynthesis and signaling (GA, ABA, auxin, ethylene), including four GA pathway genes (*FveGA20ox4*, *FveGA2ox1*, *FveGA2ox4*, and *FveGA2ox6*), as well as the ABA receptor‐encoding gene *PYRABACTIN RESISTANCE‐Like4* (*FvePYL4*); and the leucine‐rich repeat receptor‐like protein kinases, *PHLOEM INTERCALATED WITH XYLEM‐LIKE* (*FvePXL1* and *FvePXL2*), which regulate vascular‐tissue development in the stem (Figs 3e, 4a; Table S2).

To identify genes positively associated with AXB determination into BC, we next focused on the 390 DEGs of Cluster 3 whose transcript abundances were higher in *FT3* ^*OE*^ and RdV (BC) than in H4 (stolon). GO enrichment analysis indicated that it was enriched in ‘phloem development’, ‘transport’ and ‘carbohydrates’ terms, thus highlighting the importance of the vascularization process and the requirement for energy in the BC formation (Fig. 3e; Table S3). Among Cluster 3, 270 DEGs were common between *FT3* ^*OE*^ vs H4 and RdV vs H4 comparisons (Fig. 3e; Table S2). Common DEGs included transcription factors potentially involved in the regulation of meristem activity like *PISTILLATAb* (*FvePIb*), *FveMYB117b*, *YABBY2* (*FveYAB2*), *SHOOT MERISTEM LESS* (*STM*) and four *AP2/B3‐like* genes; genes related to hormone (auxin, cytokinin, GA, ethylene) biosynthesis and signaling, like the cytokinin pathway genes *CYTOKININ OXIDASE/DEHYDROGENASE* (*FveCKX4*) and *LONELY GUY3* (*FveLOG3*), and the GA pathway genes *FveGA20ox1* and *GA INSENSITIVE DWARF1* (*FveGID1*); and genes related to vascular development like two *PHLOEM PROTEIN* and two *XYLEM NAC DOMAIN* (Fig. 4a; Table S2). Altogether, these findings reflect the early specialization of AXBs which, depending on their fate as stolons or BCs, exhibit contrasted transcriptomes already at the undifferentiated stage.

### Identification of key genes involved in the regulation of branch crown differentiation by *FveFT3*

To further explore the role of *FveFT3*, we then analyzed Clusters 1 and 4 (Fig. 3f). Overexpression of *FveFT3* in H4 provokes a bushy plant phenotype because *FveFT3* promotes AXB differentiation into a BC at the expense of stolon production, likely through the reprogramming of AXB (Gaston *et al*., 2021). Moreover, in *FT3* ^*OE*^, the dormant bud (nonextended BC) stage is by‐passed so that BCs develop rapidly from AXBs (Fig. 1q). GO enrichment analysis revealed that Cluster 1 (overexpression in *FT3* ^*OE*^) is enriched in regulation and morphogenesis‐related terms, including ‘regulation of transcription’ and ‘multicellular organism development’ and Cluster 4 (downregulation in *FT3* ^*OE*^) in the ‘oxidation–reduction’ term (Fig. 3f; Table S3). To select genes strictly linked to *FveFT3* overexpression, we further examined DEGs common to the *FT3* ^*OE*^ vs H4 and *FT3* ^*OE*^ vs RdV pairwise comparisons and found 56 and 38 common DEGs in Clusters 1 and 4, respectively (Fig. 3f; Table S2). Interestingly, a large number of meristem‐related transcription factors were upregulated in *FT3* ^*OE*^ (Cluster 1), including flowering‐related genes, such as *LEAFY* (*FveLFY*), *FLOWERING LOCUS D* (*FveFD*) and five MADS‐box family genes like *FRUITFULL* (*FveFUL*), *APETALA1* (*FveAP1*) and *AGAMOUS‐like* (*AGL8*, *AGL6* and *AGL3*). Most of these MADS‐box genes showed very high transcript abundance in *FT3* ^*OE*^, the log2FC being, for example, higher than 10 for *FveFUL* in the *FT3* ^*OE*^ vs RdV comparison. Other meristem‐related transcription factors were upregulated in *FT3* ^*OE*^ (Cluster 1), such as two *YABBY* genes (*FveYAB5* and *FveYAB1a*), whereas *WUSCHELb* (*FveWUSb*) was downregulated (Cluster 4; Fig. 3f; Table S2). Upregulated hormone‐related genes included ethylene‐responsive transcription factor *Redox‐Responsive Transcription Factor 1* (*RRTF1*), *Auxin Response Factor 1* (*FveARF1*) and cytokinin‐related gene *LONELY GUY 9* (*FveLOG9*), while downregulated hormone‐related genes included two *INDOLE‐3‐ACETIC ACID* genes (*IAA2 and IAA4*) and the GA catabolism gene *FveGA2ox2* (*GA2 oxidase2*). These findings indicate that *FveFT3* overexpression not only triggers the expression of genes involved in the meristem activity necessary for transition to BC but also in flowering, which occurs much later.

### Identification of key genes involved in the induction of AXB dormancy

To identify genes possibly involved in the induction of AXB dormancy, we focused on genes differentially expressed between *FT3* ^*OE*^ and RdV, both genotypes exhibiting a BC fate, without or with a dormancy period, respectively. DEGs negatively regulated in *FT3* ^*OE*^ vs RdV are likely to be associated with AXB dormancy induction, while those positively regulated could be involved in AXB activation and outgrowth into BC (B2 to B3 stage; Figs 1q, 4a). We chose to focus on genes with known roles in shoot branching/dormancy induction (Rameau *et al*., 2015; Wang *et al*., 2018; Barbier *et al*., 2019). Not surprisingly, *FveBRC1*, known as a major repressor of shoot branching and promoting AXB dormancy in other species (Wang *et al*., 2019), was downregulated in *FT3* ^*OE*^. Downregulated DEGS also included hormone‐related genes involved in ABA biosynthesis and signaling (*NINE‐CIS‐EPOXYCAROTENOID DIOXYGENASE 3*, *NCED3; HOMEOBOX PROTEIN 40*, *HB40*), cytokinin biosynthesis (*ISOPENTENYL TRANSFERASE*, *IPT3*; CYTOKININ OXIDASE/DEHYDROGENASE, *CKX; CYTOCHROME P450*, *CYP735A1*), auxin signaling (*INDOLE‐3‐ACETIC ACID*, *IAA3 and IAA7*) and strigolactone biosynthesis (*MORE AXILLARY BRANCH*, *MAX1*). Fewer genes including the auxin biosynthesis genes *YUCCA* (*YUC6 and YUC10*) and the *ABA‐IMPORTING TRANSPORTER* (*NRT1.2/AIT1*) were upregulated in *FT3* ^*OE*^. These findings, which indicate the downregulation in *FT3* ^*OE*^ of *FveBRC1* and its downstream targets, such as HB40 and NCED3 (González‐Grandío *et al*., 2017), highlight the prominent role likely played by *FveBRC1* in regulating strawberry AXB dormancy and activation. Remarkably, *FveBRC1*, which belongs to Cluster 5, is less expressed in *FT3* ^*OE*^ and RdV than in H4 (Fig. 4a), suggesting that *FveBRC1* represses BC development, first by determining AXB fate and, later, by regulating AXB outgrowth into BC.

To deepen our understanding of the molecular mechanisms involved in AXB dormancy and activation to form a BC, we next performed the systematic analysis of transcriptomic data of the three genotypes by GSEA. To this end, we used two gene sets (‘Dormancy’ and ‘Bud activation’) previously associated with AXB dormancy and activation in Arabidopsis and potato (Nicolas *et al*., 2022; Table S4). The genes upregulated in RdV vs *FT3* ^*OE*^ AXBs were enriched in dormancy marker genes (NES = 1.49). This was also observed, to a lesser extent, in RdV vs H4 AXBs, which develop into stolon without a dormancy period (NES = 1.44). In addition, genes downregulated in RdV vs H4 were slightly enriched in bud activation markers (NES = −1.24; Fig. 4b; Table S5). These results point to the induction of dormancy in undifferentiated AXBs of RdV, but not of H4 and *FT3* ^*OE*^, and to genes likely involved in the control of this process in strawberry.

As the regulatory network controlling AXB dormancy and lateral branch outgrowth converges on BRC1 (González‐Grandío *et al*., 2013, 2017; Barbier *et al*., 2019; Wang *et al*., 2019), we further performed GSEA using BRC1‐dependent gene sets either upregulated (BRC1up) or downregulated (BRC1down) in the dormant AXB of Arabidopsis (González‐Grandío *et al*., 2013; van Es *et al*., 2024). Enrichment analysis indicated that BRC1up genes, for example, *HB40* and *NCED3*, were also upregulated in RdV (dormant AXB) compared with H4 and *FT3* ^*OE*^ (Fig. 4b; Table S5) while BRC1down genes, for example, ribosomal proteins S15 and S21 and fructokinase‐like 1, were also downregulated in RdV compared with H4 and *FT3* ^*OE*^ (Table S5). These results, which are consistent with the known function of *BRC1* in Arabidopsis, highlight the role of *FveBRC1* in the control of strawberry AXB dormancy, through the induction of genes involved in hormone signaling and the repression of genes related to protein synthesis and metabolic activity (González‐Grandío *et al*., 2017).

In addition, GSEA uncovered the previously unknown link of *FveBRC1* with AXB fate. In fact, when comparing *FT3* ^*OE*^ to H4, which present distinct fates without dormancy period, GSEA showed no enrichment in gene set related to AXB dormancy or activation but surprisingly an enrichment in the downregulated genes of the two BRC1‐dependent gene sets (BRC1up and BRC1down) in *FT3* ^*OE*^ (Fig. 4b; Table S5). Interestingly, the expression level of most BRC1down genes was higher in H4 (stolon outgrowth) than in *FT3* ^*OE*^ (BC outgrowth; Fig. 4b; Table S5). Also unexpected is the high expression level of several BRC1up genes in H4 AXBs which will produce stolons without any dormancy stage (Fig. 4b; Table S5), suggesting that BRC1 plays a prominent and hitherto unknown role in the regulation of AXB differentiation into stolon.

### *FveBRC1* contributes to AXB fate determination and dormancy in strawberry

**Fig. 5:**
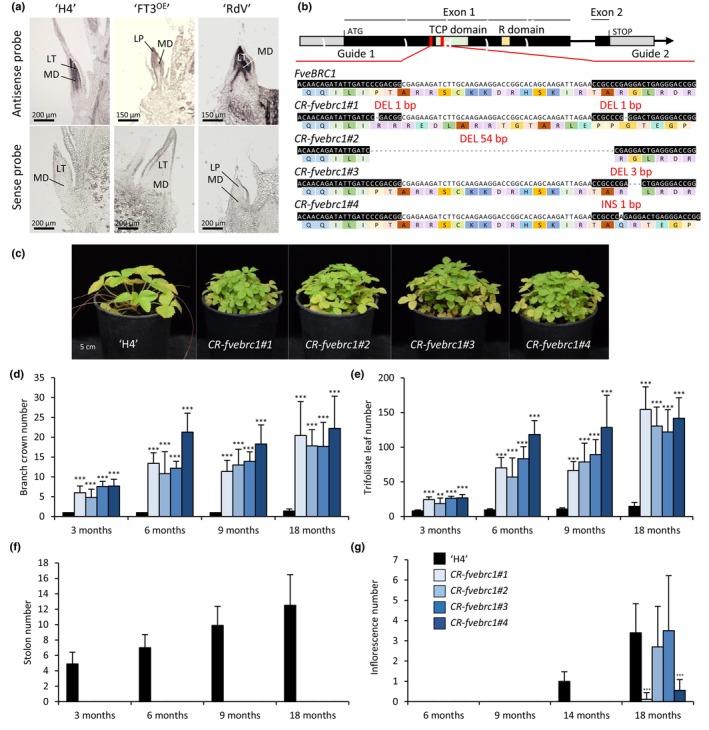
*FveBRC1* modulates plant architecture by acting on axillary bud (AXB) fate determination and AXB outgrowth in strawberry. (a) *In situ* hybridization of *FveBRC1* on undifferentiated AXBs of four‐leaf stage plants of three genotypes ‘Hawaii‐4’ (H4), *35S::FveFT3* ^*FveOE*^ (*FT3* ^*OE*^) and ‘Reine des Vallées’ (RdV). LP, leaf primordium; LT, leaflet; MD, meristematic dome. (b) CRISPR/Cas9‐induced mutations in exons of *FveBRC1* produced four independent mutations. Red lines indicate the two guide RNA target sequences. Insertions (INS) or deletions (DEL) are indicated in red. (c) Ten‐month‐old plants of four T1‐homozygous *CR‐fvebrc1* mutants with the wild‐type H4. (d–g) Bar graphs represent the number of (d) branch crown, (e) trifoliate leaf, (f) stolon and (g) inflorescence in H4 and T1‐homozygous *CR‐fvebrc1* mutants. For each genotype, 10 independent seed‐derived plants were analyzed (*n*=10; error bars = +SE). Wilcoxon‐Mann–Whitney test showed significant differences (*, *P* ≤ 0.05; **, *P* ≤ 0.01; ***, *P* ≤ 0.001) between H4 and the mutants (*n* = 10).

Given the central role of *BRC1* in repressing AXB outgrowth (Aguilar‐Martínez *et al*., 2007; Wang *et al*., 2019) and its possible role in the determination of AXB fate, which has never been investigated to date, we further explored the function of *FveBRC1* in strawberry. We examined by *in situ* hybridization the spatial accumulation of *FveBRC1* transcripts in AXBs at the four‐leaf stage in H4, *FT3* ^*OE*^ and RdV (Fig. 5a). *FveBRC1* transcripts were much less abundant in *FT3* ^*OE*^ AXBs, where BC outgrowth occurs rapidly, than in H4 AXBs, which produce stolons, and in RdV AXBs, which produce dormant AXBs. These observations reinforce the transcriptomic findings (Fig. 4a; Table S2) that a decrease in *FveBRC1* expression is associated with AXB outgrowth into BC and determination of AXB fate. To further investigate this hypothesis, we generated mutant lines using CRISPR/Cas9 gene editing in the H4 genotype by targeting the exon 1 of *FveBRC1*, which contains the TCP domain, with two guide RNAs (Fig. 5b). We selected four T1‐homozygous allelic mutants. Two mutants displayed frameshift mutations, including a deletion of one base in both guide sequences in the *CR‐fvebrc1#1* mutant, and an insertion of one base in guide 2 in the *CR‐fvebrc1#4* mutant. *CR‐fvebrc1#2* and *CR‐fvebrc1#3* mutants had a large deletion and an in‐frame 3 bp deletion, respectively. These mutants displayed a pronounced bushy phenotype with no stolon compared with H4 (WT; Fig. 5c). The bushy phenotype was due to the presence of a high number of BCs and their associated trifoliate leaves, both of which increased drastically over the 18 months of observation (Fig. 5d,e). Plant architecture analysis further showed that the modification of the BC/stolon balance is due to a change in AXB fate determination as all AXBs developed into BCs, instead of stolons as in H4 (Fig. S4). Remarkably, even the AXBs in the axils of the juvenile leaves at the first two nodes developed into BCs, whereas they remained dormant in H4 (Fig. S4). Thus, the bushy phenotype of these mutants is also due to the continuous development of BCs on the successive orders of branching (secondary, tertiary etc. BCs; Fig. S4).

**Fig. 6:**
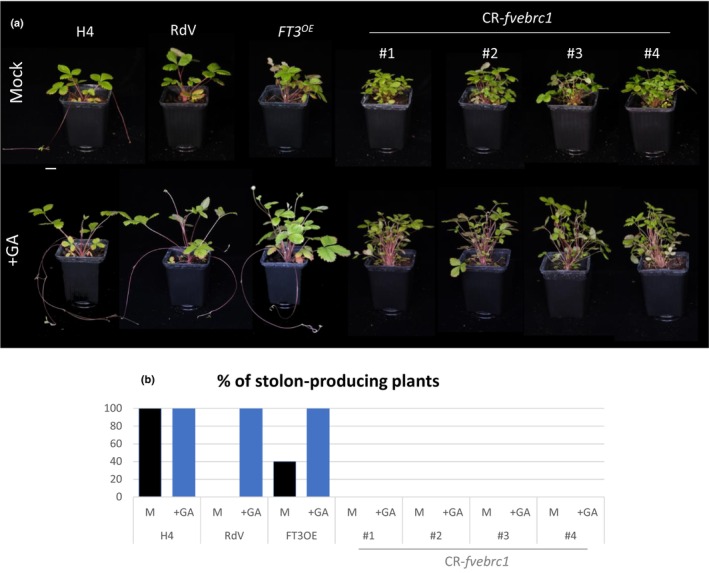
Effect of gibberellins (GA) treatment on ‘Hawaii‐4’ (H4), *35S::FveFT3* ^*FveOE*^ (*FT3* ^*OE*^), ‘Reine des Vallées’ (RdV) and CR‐*fvebrc1* strawberry genotypes. (a) Representative plants of H4, RdV, *FT3* ^*OE*^ and T2 homozygous CR‐*fvebrc1* mutants after 1 month of mock or GA treatment applied to 5‐month‐old plants. Bar, 2 cm. (b) Percentage of stolon‐producing plants for each genotype after mock (M) or GA (+GA) treatments. Mock treatment was used as a negative control. Five plants per genotype and per treatment condition were analyzed.

To further elucidate whether *FveBRC1* regulates AXB fate determination into stolon through the GA pathway, which is known to be essential for stolon formation (Tenreira *et al*., 2017), we first examined the expression of *FveGA20ox4* in the AXBs of *CR‐fvebrc1* mutants. *FveGA20ox4* expression was markedly reduced in these mutants (Fig. S5), thus suggesting that the stolon formation by *FveBRC1* can be mediated by *FveGA20ox4*. We then evaluated whether GA application could rescue stolon formation in the absence of functional *FveBRC1* by applying GA3 to *CR‐fvebrc1* mutants. GA3 treatments were also performed on H4 and RdV, used as control, and on another BC‐forming genotype *FT3* ^*OE*^ (Fig. 6). As expected from previous studies (Tenreira *et al*., 2017), all H4 plants formed stolons with and without GA3 treatment, and the runnerless RdV genotype (*ga20ox4*) only produced stolons when GA3 was applied. Under control conditions, *FT3* ^*OE*^ genotype displayed a drastic reduction in stolon compared with H4 (WT; Gaston *et al*., 2021) with only 40% of the 5‐month‐old plants producing stolons. Following GA3 treatments, all *FT3* ^*OE*^ plants formed stolons, suggesting that GA supply can compensate for the reduction in *FveGA20ox4* expression caused by *FveFT3* overexpression. By contrast, *CR‐fvebrc1* mutants failed to produce stolons even after GA3 treatment, indicating that GA alone is not sufficient for determining AXB fate, and that a functional *FveBRC1* gene is required for stolon production (Fig. 6).

## Discussion

In *Fragaria* species, AXBs located in the leaf axil on the stem (primary crown) can differentiate into either lateral shoot bearing inflorescences (BC) or specialized stems (stolons), which produce daughter plants, enabling strawberry plant multiplication. Controlling AXB fate is therefore essential, as it determines the balance between sexual and asexual reproduction, and hence the antagonistic yields of fruit and daughter plants in strawberry (Tenreira *et al*., 2017). AXB fate has been shown to be controlled by environmental signals, such as day length and temperature (Andrés *et al*., 2021; Andrés & Koskela, 2022), and by endogenous signals, including GA (Thompson & Guttridge, 1959; Hytönen *et al*., 2009; Tenreira *et al*., 2017; Caruana *et al*., 2018) and cytokinins (CK; Pritts *et al*., 1986; Li *et al*., 2021; Han *et al*., 2024). However, a comprehensive view of the determination of AXB fate at the molecular level is still lacking.

Potato, a well‐studied stolon‐forming crop species, is often used as a reference for studying AXB in strawberry (Gaston *et al*., 2020) since, depending on their position, potato AXBs have the potential to remain dormant or develop into a branch or a stolon (Nicolas *et al*., 2022). To date, major advances in potato, Arabidopsis and other plant species provided new insights into the regulation of AXB outgrowth by gene regulatory networks in response to various genetic and environmental cues (Wang *et al*., 2018; van Es *et al*., 2024). These studies have highlighted the key role of the master transcription factor *BRC1* in orchestrating AXB dormancy establishment and its subsequent release allowing AXB outgrowth into a lateral branch. However, how *BRC1* could play a role in stolon differentiation has been little studied in species with alternative AXB fate. In potato, technical limitations, that is, the current inability to predict the AXB fate (stolon or lateral branch) shortly after initiation, have so far prevented transcriptomic studies from addressing the molecular mechanisms underlying the trade‐off between stolon and lateral branch formation.

Here, in strawberry, by first predicting the AXB fate while still morphologically undifferentiated, and then using RNA‐seq on undifferentiated AXBs from three strawberry genotypes with contrasted stolon or BC production, we were able to: uncover transcriptional signatures associated with different AXB fates; demonstrate that undifferentiated AXB can already recapitulate, at least partially, the developmental program leading to stolon production or BC formation and flowering; and show that BRC1 not only orchestrates AXB dormancy/activation for BC production, as in other plant species studied to date, but also likely plays a hitherto unknown role in AXB fate determination in stolon. This study provides a comprehensive view of the molecular control of AXB fate in strawberry. It further highlights that *FveBRC1* represses BC development not only by inhibiting AXB outgrowth into BC, as observed in many plant species, but also by acting upstream, through the determination of AXB fate.

### The fate of undifferentiated AXBs can be predicted according to their node position on primary crown

Starting from undifferentiated AXBs, we first described their developmental trajectories according to their differentiation into either stolons or BCs (Fig. 1), as previously done for strawberry SAM (Jahn & Dana, 1970; Gaston *et al*., 2021) and, more recently, AXBs (Han *et al*., 2024). We showed that these developmental trajectories vary as a function of genotype and node position on the stem, consistent with our previous study of octoploid strawberry (Labadie *et al*., 2023a). We observed that AXBs producing stolons soon undergo basal elongation, in agreement with previous findings (Savini *et al*., 2005; Han *et al*., 2024). By contrast, AXBs producing BCs enlarge as their first leaves emerge, followed by a period of AXB dormancy (Labadie *et al*., 2023a; Han *et al*., 2024). Dormant AXBs, which develop into BCs (Han *et al*., 2024), eventually burst after a lag period of variable length. In *FT3* ^*OE*^, a distinct H4‐derived genotype producing BCs, BC formation occurs without AXB dormancy (Gaston *et al*., 2021), suggesting very early AXB activation. Thanks to this founding work on three genotypes with distinct plant architectural behaviors, producing either stolons (H4), BCs (RdV) or BCs without an intermediate AXB dormancy stage (*FT3* ^*OE*^; Gaston *et al*., 2021), we were able to predict the fate of a given undifferentiated AXB with high confidence.

### Undifferentiated AXBs already display transcriptional signatures related to their fate

A major finding of our study is that transcriptional signatures of undifferentiated AXBs are already very contrasted depending on their fate, despite the absence of visible morphological changes at this developmental stage. The most striking example is *FT3* ^*OE*^ where the gene expression signature of the undifferentiated AXB already includes a large number of highly expressed flowering‐associated genes, such as *FveFUL*, *FveLFY*, *FveFD* and *FveAP1* (Freytes *et al*., 2021), but low expression of dormancy‐associated genes, including the ABA biosynthesis and signaling genes *NCED3* and *HB40* (González‐Grandío *et al*., 2017). This pattern effectively recapitulates the progression of BC differentiation and development in this genotype. Conversely, the undifferentiated AXB of the BC‐forming genotype RdV, which will undergo dormancy after bud enlargement, already exhibits high expression of dormancy‐related genes. These include the signal integrator *FveBRC1*, which promotes AXB dormancy, the ABA‐related genes *NCED3* and *HB40*, and the strigolactone biosynthesis gene *MAX1*, which reinforces establishment of bud dormancy by enhancing *BRC1* activity (Gomez‐Roldan *et al*., 2008; Kelly *et al*., 2023; van Es *et al*., 2024). Despite the well‐established role of strigolactones in regulating AXB shoot dormancy via *BRC1* (Khuvung *et al*., 2022; Kelly *et al*., 2023), the genes involved in strigolactone biosynthesis are underrepresented in our study as well as in two other studies on AXB outgrowth in strawberry (Qiu *et al*., 2019; Sun *et al*., 2025). Additionally, the *FveMYB117a*‐regulated *CKX1* gene, which represses cytokinin accumulation and thereby prevents AXB outgrowth to BC (Han *et al*., 2024), is highly expressed in RdV AXB where it probably impedes AXB activation.

The distinction between transcriptional signatures associated with either BC or stolon fates is more complex, as the formation of both requires meristem differentiation and vascular development, which are regulated by various hormones (GA, cytokinin, auxin, ethylene and brassinosteroid) and sugars (Barbier *et al*., 2019; Aloni, 2021; Guo *et al*., 2021; Yuan *et al*., 2024). Nevertheless, cluster analysis highlighted genes more specifically linked to either stolon or BC fate, most of which have unknown roles in strawberry (Fig. 3). Notably, the *FveZFP6* gene, which was recently identified as a key regulator of the stolon fate determination through the activation of the GA pathway, was detected in our cluster analysis, thereby validating the relevance of our results (Guo *et al*., 2026). Among the meristem‐related TFs strongly upregulated in stolon‐forming AXBs is *FveBFT*, a homolog of *FveTFL1*, a systemic long‐day antiflorigen contributing to photoperiodic regulation of flowering in strawberry (Li *et al*., 2019a; Gaston *et al*., 2021). Many more meristem‐related TFs are markers of BC‐forming AXBs, including several AP2/B3‐like TFs. Among them, two members (*FvH4_1g14660* and *FvH4_1g14680*) are homologous to the Arabidopsis *VERNALIZATION1* (VRN1) gene, which represses the floral repressor *FLOWERING LOCUS C* (*FLC*) during vernalization, thus accelerating floral transition (Levy *et al*., 2002). *FveSTM‐like* is homologous to Arabidopsis *STM*, a KNOTTED1‐like homeobox (KNOX) gene essential for AXM initiation, acting through meristem maintenance and hormonal regulation (Hay & Tsiantis, 2010; Lechon *et al*., 2025). *FveYAB2* and *FvePIb* are homologous to Arabidopsis *YABBY* and *PISTILLATA* genes, which regulate abaxial cell fate and laminar outgrowth (Siegfried *et al*., 1999), and petal and stamen specification (Goto & Meyerowitz, 1994), respectively. Their functions are consistent with their possible role in shoot and inflorescence development in BC. We also identified the MYB TF *FveMYB117b* (*FvH4_5g27870*) as a marker of BC fate, with similar expression in *FT3* ^*OE*^ and RdV AXBs. *FveMYB117b* is a close homologous of *FveMYB117a* (*FvH4_4g31190*), recently identified as a key repressor of AXB outgrowth for BC formation (Han *et al*., 2024). However, CRISPR‐editing of *FveMYB117b* had no discernible effect on AXB outgrowth, leaving its precise role in AXB fate determination unresolved (Han *et al*., 2024).

Genes involved in hormonal biosynthesis and signaling are well represented in the cluster analysis associated with AXB fate, as expected given their role in the regulation of shoot branching (Barbier *et al*., 2019; Yuan *et al*., 2023). To date, in strawberry AXB, GA has been primarily linked to stolon formation (Tenreira *et al*., 2017; Caruana *et al*., 2018; Andrés *et al*., 2021), auxin and ABA to AXB dormancy (Qiu *et al*., 2019) like in Arabidopsis and potato (González‐Grandío *et al*., 2017; Nicolas *et al*., 2022), and CK to BC outgrowth (Qiu *et al*., 2019; Han *et al*., 2024). Among the AXB fate‐related clusters, several genes encode GA 20‐oxidases and GA 2‐oxidases. Bioactive GAs that promote organ differentiation and growth are synthesized in meristem primordia through the key GA biosynthetic enzyme GA 20‐oxidase (Tenreira *et al*., 2017). To maintain meristem indeterminacy, GA 2‐oxidase converts bioactive GAs into inactive forms, preventing their activity in the meristematic dome (Galinha *et al*., 2009; Veit, 2009; Andrés *et al*., 2014; Tenreira *et al*., 2017). As expected, *FveGA20ox4*, which, when inactivated, leads to runnerless phenotype (Tenreira *et al*., 2017), was highly expressed in stolon‐forming AXBs, confirming the prominent role of this specific GA20 oxidase in stolon formation. The function of GA in stolon and BC outgrowth appears more complex, as several GA 20‐oxidases and GA 2‐oxidases are differentially expressed in AXBs with different fates, suggesting that their specific actions depend on the precise timing and localization of their expression. Unexpectedly, BC‐forming AXBs exhibited higher expression of a GA receptor, *GID1B‐like*, known to target DELLA for degradation (Hauvermale *et al*., 2012), whereas the absence of the DELLA protein, FveRGA1, leads to stolon formation in a *runnerless* strawberry genotype (Caruana *et al*., 2018).

Like GAs, CKs are key regulators of plant growth and development, particularly in cell divisions. In AXB, CKs primarily regulate BC outgrowth (Waldie & Leyser, 2018; Yuan *et al*., 2023; Han *et al*., 2024). CK levels are fine‐tuned by cytokinin oxidase (CKX) genes, which catalyze CK degradation (Schmülling *et al*., 2003) and are differentially expressed in stolon‐ vs BC‐forming AXBs (Fig. 4; Table S2). The high expression of a *LOG* gene, *FveLOG3*, in AXBs of BC‐forming genotypes suggests that cytokinin activation is required not only for BC outgrowth (Han *et al*., 2024) but also at an earlier stage, *for example* at the bud enlargement (B1) stage.

**Fig. 7:**
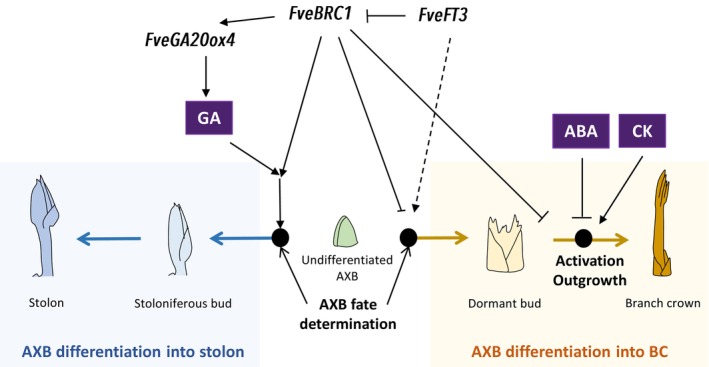
Proposed regulatory model for the axillary bud (AXB) fate determination of outgrowth into stolon and branch crown (BC) in strawberry. *FveBRC1* regulates the activity of *FveGA20ox4* and, through a complementary pathway, they act together for the AXB fate determination into stolon. *FveFT3* influences AXB fate determination, notably by repressing *FveBRC1*. Simultaneously, *FveBRC1* negatively regulates AXB fate determination into BC and AXB outgrowth. Abscisic acid (ABA) inhibits, whereas cytokinin (CK) promotes BC outgrowth. Arrows indicate positive regulation, bars indicate repression, and dotted arrows indicate hypothetical or partially supported regulatory relationships.

These findings highlight the early specialization of undifferentiated AXBs. Although no morphological changes are visible at this stage, a specific developmental trajectory is already programmed through a distinct network of transcription factors and hormones cross‐talk. This regulatory network likely plays a crucial role in AXB fate determination, with GA playing a prominent role in AXB differentiation into a stolon while CK and ABA influence AXB differentiation into a BC (Fig. 7).

### FveBRC1 controls not only AXB outgrowth into branch crowns but also AXB fate in strawberry

Our study, based on both transcriptomic analysis of undifferentiated AXBs and functional validation *in planta*, confirms that *FveBRC1* induces AXB dormancy and represses shoot branching in strawberry, supporting previous indications on its role in BC outgrowth in the strawberry *FveMYB117a* mutant (Han *et al*., 2024). The role of BRC1 as a key integrator of apical dominance and hormone signaling in the control of shoot architecture, primarily through the repression of AXB outgrowth, has already been demonstrated in many plant species (Aguilar‐Martínez *et al*., 2007; Poza‐Carrión *et al*., 2007; Braun *et al*., 2012; Muhr *et al*., 2018; Wang *et al*., 2019). In the same way, in strawberry, *FveBRC1* has been shown to be negatively regulated by CK and positively by auxin, strigolactone and ABA (Han *et al*., 2024; Sun *et al*., 2025). An original finding of our study is that *FveBRC1* likely fulfills an additional role in strawberry by contributing to AXB fate determination. In fact, *FveBRC1* mutation on H4 genotype not just leads to bud dormancy release but also leads to a runnerless phenotype. This reprogramming of AXB fate indicates that *FveBRC1* participates in AXB fate determination in addition to this role in AXB outgrowth in BCs. Such a role has been proposed in potato, where the two paralogs *StBRC1a* and *StBRC1b* control stolon/tuberization and branch formation (Nicolas *et al*., 2015, 2022). This raises the question: how does *FveBRC1* regulate AXB fate determination into stolon in *F. vesca*?

*Fve*BRC1 may regulate AXB fate determination through modulation of the GA pathway. BRC1 regulation of GA pathway has been already observed in Arabidopsis, where various GA‐associated genes are directly regulated by *AtBRC1* (van Es *et al*., 2024) and, in potato, where the *StAST1* TCP gene induces the expression of *StGA20ox1* during tuberization (Sun *et al*., 2024). In *F. vesca*, the GA pathway genes *FveGA20ox4* and *FveRGA1* play key roles in stolon fate determination (Tenreira *et al*., 2017; Caruana *et al*., 2018; Li *et al*., 2018). In this study, we observed a reduced expression of *FveGA20ox4* in the *CR‐fvebrc1* mutant, suggesting that *FveBRC1* may act as a positive regulator of *FveGA20ox4* transcription. However, in the *CR‐Fvebrc1* mutant, exogenous GA application failed to rescue stolon formation. We therefore propose a model in which AXB fate is determined by the combined action of *FveBRC1* and GA in strawberry. When *FveBRC1* is functional, AXB fate is GA‐dependent and sufficient GA levels promote stolon formation (*e.g*. H4), whereas GA deficiency reprograms AXBs toward the formation of BC (e.g. RdV). By contrast, when *FveBRC1* is nonfunctional, AXB fate is uncoupled from GA status and is always reprogrammed toward BC formation (e.g. in the *CR‐fvebrc1* mutants), even in the presence of functional *FveGA20ox4* or exogenous GA application. This indicates that functional *FveBRC1* and GA are both necessary for stolon production (Fig. 7).

*FveBRC1* may also influence AXB fate determination by interacting with *FveFT3*, which promotes the formation of BCs instead of stolons (Gaston *et al*., 2021). Protein–protein interactions between members of BRC1 and FT families have been shown in various species (Niwa *et al*., 2013; Maurya *et al*., 2020; Feng *et al*., 2022; Nicolas *et al*., 2022), which supports antagonistic interactions among them. For example, in potato, StBRC1B induces AXB dormancy by interacting with the tuberigen protein SP6A, an FT homolog, thereby inhibiting tuber induction in aerial nodes (Navarro *et al*., 2011; Nicolas *et al*., 2022). In strawberry, the antagonistic relationship between *FveFT3* and *FveBRC1* is supported by the production of BCs instead of stolons in both *FT3* ^*OE*^ (Gaston *et al*., 2021) and *CR‐fvebrc1* mutants (Fig. 6). Moreover, we showed that *FveFT3* overexpression leads to a reduction in *FveBRC1* expression (Figs 4, 7). However, as the production of stolons is not completely prevented in *FT3* ^*OE*^ plants, which are still able to produce stolons after a delay (Gaston *et al*., 2021), these results suggest that *FveBRC1* affects stolon formation in a dose–response relationship. Importantly, as exogenous GA application increases stolon formation in *FT3* ^*OE*^ plants, GA may compensate for the reduced *FveGA20ox4* expression in *FT3* ^*OE*^ (Fig. 4). Overall, these results support a model in which *FveFT3* acts to repress *FveBRC1*, which in turn regulates GA biosynthesis via *FveGA20ox4*, and ultimately influences the fate of AXB (Fig. 7).

In conclusion, our findings indicate that AXB fate is established very early after AXB initiation and provide gene targets for studying the developmental processes underlying stolon and BC formation as well as their regulation by various endogenous cues, including the BRC1 repressor. Our study and published results suggest that BRC1 not only plays a central function as a negative regulator of AXB branch outgrowth, by keeping AXB dormant, but also plays at the same time an essential but less well‐established role in regulating AXB fate determination (Fig. 7). Future developments will take advantage of our large gene expression dataset to explore the mechanisms involved in AXB differentiation, in particular the novel and unique function of *FveBRC1* in stolon fate determination. From a more applied perspective, our results highlight *FveBRC1* as a regulator of the balance between sexual and asexual reproduction in strawberry, warranting further investigation in cultivated octoploid strawberry.

## Competing interests

None declared.

## Author contributions

MA, BD and AG conceived the study and designed experiments. MA organized experiments. MA, PP, AP and MH realized experiments. MA realized the histological, statistical and bioinformatic analyses with the help of YC and PM. AG, BD, CR and MA wrote the manuscript with the help of MN. All the authors discussed the results and commented on the manuscript.

## Disclaimer

The New Phytologist Foundation remains neutral with regard to jurisdictional claims in maps and in any institutional affiliations.

## Supporting information


**Fig. S1** ‘Hawaii‐4’, *35S::FveFT3* ^*FveOE*^ and ‘Reine des Vallées’ plants at the five‐leaf stage.
**Fig. S2** Morphological characterization of AXB development into a branch crown in ‘Reine des Vallées’.
**Fig. S3** Phylogenetic analysis of TCP proteins.
**Fig. S4** Schematic representation of the architecture of 4‐month‐old ‘Hawaii‐4’ and *CR‐fvebrc1* T1 plants.
**Fig. S5** Expression pattern of *FveGA20ox4* in *CR‐fvebrc1#2* undifferentiated AXBs.

**Table S1** List of primers used in the manuscript.
**Table S2** Results of RNA‐seq analyses.
**Table S3** Significant Gene Ontology categories (*P*‐value < 0.05) for each cluster.
**Table S4** Gene sets used for Gene Set Enrichment Analysis.
**Table S5** Gene Set Enrichment Analysis results for each pairwise comparison.
Please note: Wiley is not responsible for the content or functionality of any Supporting Information supplied by the authors. Any queries (other than missing material) should be directed to the *New Phytologist* Central Office.

## Data Availability

The transcriptomic data discussed in this publication have been deposited in NCBI's Gene Expression Omnibus (Edgar *et al*., 2002) and are accessible through GEO Series accession no.: GSE 292941 (https://www.ncbi.nlm.nih.gov/geo/query/acc.cgi?acc=GSE292941).
