# Supplementary material for: The molecular regulation of axillary bud fate determination and outgrowth into branch crown in strawberry involves BRC1
Source: New Phytol. 2026 Jun 26;251(5):3021–38. doi: 10.1111/nph.71375 (PMC13443198; doi:10.1111/nph.71375)

New Phytologist

New Phytologist Supporting Information

Article title: **The molecular regulation of axillary bud fate determination and outgrowth into branch crown in strawberry involves _BRC1_**

Authors: Marie Alonso, Pierre Prévost, Aline Potier, Pascal GP Martin, Yves Caraglio, Michael Nicolas, Michel Hernould, Christophe Rothan, Béatrice Denoyes, and Amèlia Gaston

Article acceptance date: 01 June 2026

The following Supporting Information is available for this article:

Fig. S1 'Hawaii-4' (H4), *35S::FveFT3*^{FveOE} (*FT3*^{OE}) and 'Reine des Vallées' (RdV) plants at the 5-leaf stage.

Fig. S2 Morphological characterization of AXB development into a branch crown in 'Reine des Vallées' (RdV).

Fig. S3 Phylogenetic analysis of TCP proteins.

Fig. S4 Schematic representation of the architecture of 4-month-old 'Hawaii-4' (H4) and *CR-fvebrc1* T1 plants.

Fig. S5 Expression pattern of *FveGA20ox4* in *CR-fvebrc1#2* undifferentiated AXBs.

Table S1 List of primers used in the manuscript.

Table S2 Results of RNAseq analyses.

Table S3 Significant Gene Ontology (GO) categories (p-value < 0.05) for each cluster.

Table S4 Gene sets used for gene set enrichment analysis (GSEA).

Table S5 Gene Set Enrichment Analysis (GSEA) results for each pairwise comparison.

Fig. S1 'Hawaii-4' (H4), *35S::FveFT3Fve^{OE}* (*FT3^{OE}*) and 'Reine des Vallées' (RdV) plants at 5-leaf-stage. Numbers indicate the order of emergence of the leaves (1 = the oldest, 5 = the most recent). The white arrow shows the emerging leaf where the undifferentiated axillary bud is located. The orange arrow shows stolons in H4. Bar = 1 cm.

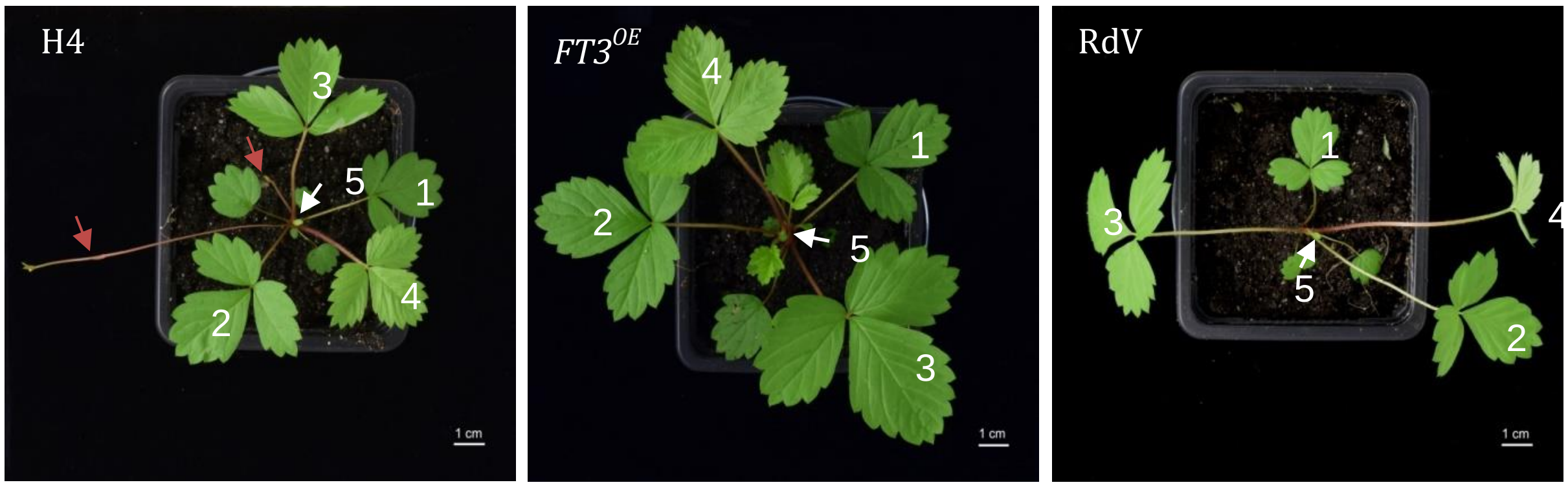

Fig. S2 Morphological characterization of AXB development into a branch crown in 'Reine des Vallées' (RdV). Bar = 0.5 mm.

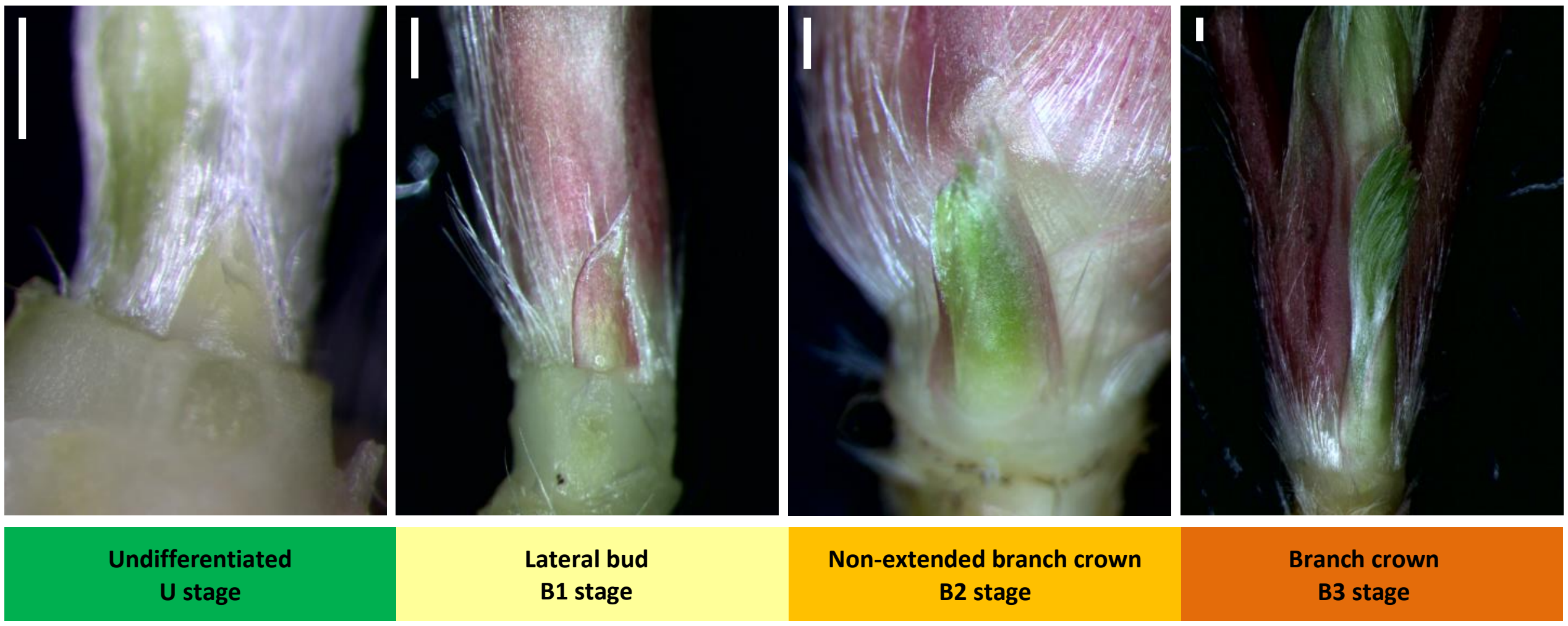

Fig. S3 Phylogenetic analysis of TCP proteins: FveTCPs from *Fragaria vesca*, AtTCPs from *Arabidopsis thaliana*, TEOSINTE BRANCHED1 (TB1) from *Zea mays*, CYCLOIDEA (CYC) from *Antirrhinum majus*, and PROLIFERATING CELL FACTOR 1 and 2 (PCF1 and PCF2) from *Oryza sativa*. The phylogenetic tree was constructed from the alignment of the amino acid sequences of the proteins by neighbor-joining method using Geneious Prime. Numbers at nodes indicate bootstrap support values based on 1000 replicates. FveTCP proteins are marked in bold. Colored areas indicate different TCP subgroups (orange = CYC/TB1, blue = CIN, red = PCF).

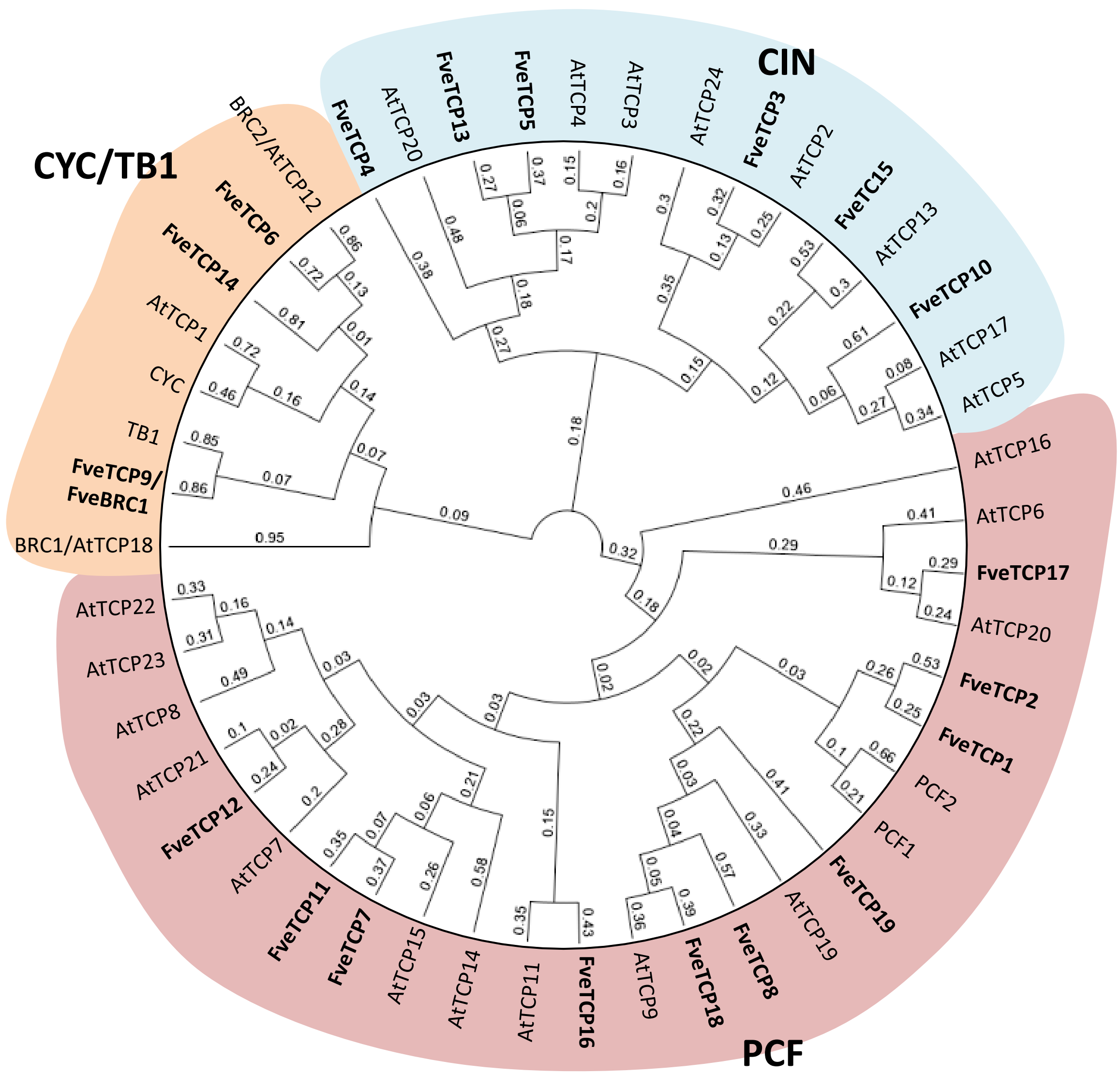

Fig. S4 Schematic representation of the architecture of 4-month-old 'Hawaii-4' (H4) and CR-fvebrc1 T1 plants. On each primary crown (vertical green line), leaves present at their axil an axillary bud (AXB) that can either remain latent (green dot), give a stolon (blue arrow) or give a branch crown (BC) (pink arrow).

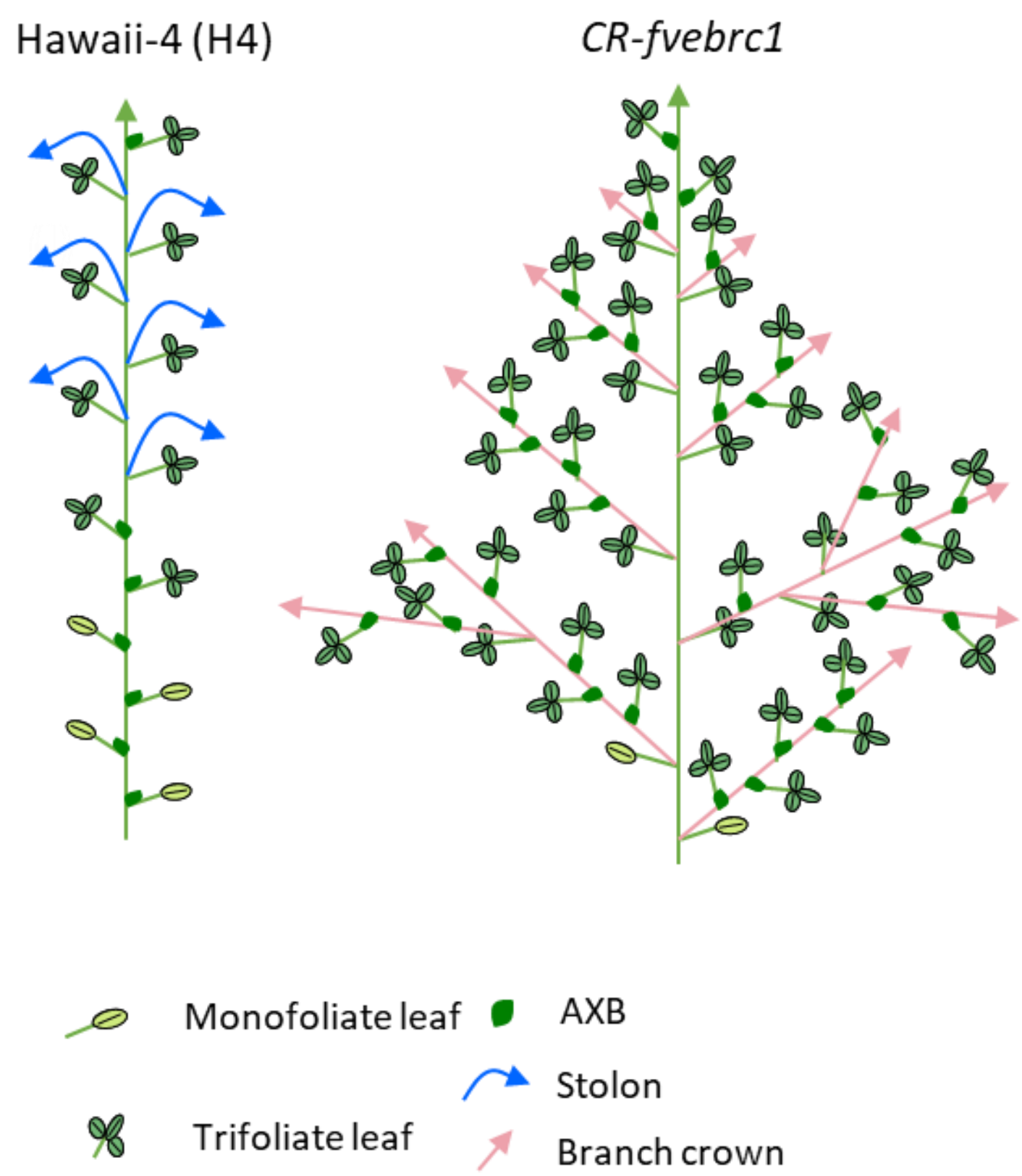

Fig. S5 Expression pattern of *FveGA20ox4* in *CR-fvebrc1#2* undifferentiated AXBs. Error bars, mean ± SE (*n* = 3 biological replicates of 30 undifferentiated AXBs), with transcripts normalized to *FveMSI*. A Kruskal–Wallis test (ANOVA on the rank and appropriate post hoc test) was used to highlight significant difference.

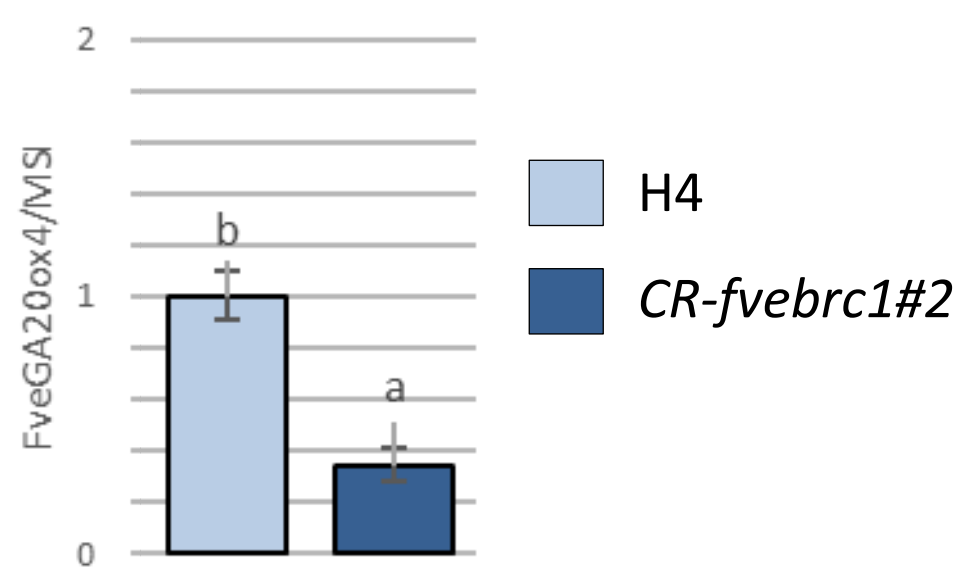

Supplement: Supplementary file 1 [file NPH-251-3021-s001.pdf]
